# Differentially Regulated Apolipoproteins and Lipid Profiles as Novel Biomarkers for Polypoidal Choroidal Vasculopathy and Neovascular Age-Related Macular Degeneration

**DOI:** 10.3389/fendo.2022.946327

**Published:** 2022-07-19

**Authors:** Xinyuan Zhang, Bingjie Qiu, Zhizhong Gong, Xiaosi Chen, Yanhong Wang, Yao Nie

**Affiliations:** ^1^ Beijing Tongren Eye Center, Beijing Tongren Hospital, Capital Medical University, Beijing, China; ^2^ Beijing Retinal and Choroidal Vascular Disorders Study Group, Beijing, China; ^3^ Division of Medical Affairs, Beijing Hospital of Traditional Chinese Medicine, Capital Medical University, Beijing, China; ^4^ Department of Epidemiology and Biostatistics, Institute of Basic Medical Sciences Chinese Academy of Medical Sciences & School of Basic Medicine Peking Union Medical College, Beijing, China

**Keywords:** polypoidal choroidal vasculopathy, neovascular age-related macular degeneration, serum lipid profiles, apolipoproteins, regulatory mechanisms

## Abstract

Lipid dyshomeostasis has been implicated in the pathogenesis of various retinal and choroidal vascular diseases. This study aims to investigate whether apolipoprotein (apo) mediated differential regulation of lipid metabolism contributes to the phenotypes of polypoidal choroidal vasculopathy (PCV) and neovascular age-related macular degeneration (nAMD). This study involved 148 subjects including 53 patients with PCV, 44 patients with nAMD, and 51 age-, sex-matched subjects with normal fundus controls. Routine blood biochemistry profile was evaluated. Apolipoproteins was estimated by Luminex technology. After controlling for age, gender, body mass index, duration of hypertension and type 2 diabetes mellitus, apoB/non-high density lipoprotein cholesterol (HDL-C) (*p*=0.015) was an independent risk factor for nAMD, apoB was an independent risk factor for PCV(*p*=0.011), compared with control. Low-density lipoprotein cholesterol (LDL-C) was significantly higher in patients with PCV when compared with nAMD (*p*=0.037). Furthermore, apoB/non-HDL, LDL-C, triglycerides and were significantly correlated with the pathogenesis of subgroups of PCV and nAMD. We concluded that lipid profiles and apos are differential regulated in PCV, nAMD and their subtypes, indicating different pathogenicity contributed to the different phenotypes of PCV and nAMD. Non-pachy PCV shares pathological similarities with nAMD, which is highly correlated with age-related atherosclerosis.

## Introduction

Age-related macular degeneration (AMD) is a major and irreversible blindness in elderly people worldwide (1, 2). Polypoidal choroidal vasculopathy (PCV), which is considered a distinct subtype of neovascular AMD (nAMD), is more prevalent in Asia population than Caucasians although it was first reported by Dr. Yannuzzi in US in the 1990s (3, 4). Both PCV and nAMD are multifactorial disorders and share a variety of characteristics in phenotype, risk factors, natural history, and treatment strategies (5). Recently, several genetic and epigenetic studies raised the hypothesis that PCV is a genetic disorder, AMD-related gene polymorphisms such as *ARMS2* A69S and *CFH* Y402H (6) have been found associated with PCV. On the other hand, the disparity in response to intravitreal anti-VEGF drugs between nAMD and PCV has also received extensive attention. Although the natural history, clinical features, pathological and genetic correlation of the two diseases has been intensively studied (5, 7–9), the pathogenic similarities and differences between PCV and nAMD remain largely unknown.

Dysregulated lipid metabolism has been implicated in the pathogenesis of PCV and nAMD. Lipoproteins are soluble lipid protein complexes that are formed by the interactions between apolipoproteins (apos, amphipathic proteins) and lipids (10). Abnormal expression of genes of lipid metabolism and the reverse cholesterol transport (RCT) pathway, such as *Cholesterol Ester Transfer Protein (CETP)*, *Hepatic Lipase (LIPC), ATP-binding Cassette Transporter A1 (ABCA1)* and *Apolipoprotein E (APOE)*, has been reported in patients with PCV and nAMD (11–13). Furthermore, dysregulated lipoproteins [triglycerides (TG), total cholesterol (TC), high-density lipoprotein- cholesterol (HDL-C) and low-density lipoprotein (LDL-C)] and apolipoproteins have been reported in both PCV and nAMD (12, 14, 15); however, results from existing studies are controversial and the connections remain elusive (15, 16).

The differential regulation mechanism and the interactions between apos and lipid profiles remain uncertain. Our previous studies have shown that abnormal plasma levels of apolipoproteins and lipid profiles are correlated with variety of retinal vascular disorders including diabetic retinopathy (17–19). It is interesting to determine if apolipoprotein-mediated differential regulation of lipid metabolism is a pathological cause of the clinical phenotypes of nAMD and PCV. In this study, we aim to determine the differential serum levels of lipoproteins, apoproteins and their ratios, and to determine their associations with the pathogenesis of PCV and nAMD.

## Materials and Methods

### Study Subjects

The study subjects were selected from our Retinal and Choroidal Study Cohort (RCSC), a prospective cohort from year of 2016 to 2022 in Beijing Tongren Eye Center. This cohort comprises patients with PCV, nAMD, pachychoroid pigment epitheliopathy, pachychoroid neovasculopathy, focal choroidal excavation, peripapillary pachychoroid syndrome pachydrusen and age-, sex-, duration of the systemic disorders (hypertension and diabetes mellitus) matched controlled subjects (with normal fundus in both eyes). This study was approved by the institutional review board of Beijing Tongren Hospital and adhered to the tenets of the Declaration of Helsinki. All participants signed informed consent form approved by the Ethics Committee of Beijing Tongren Hospital, Capital Medical University before participation.

This nested case-control study compromised 148 subjects, including 53 patients with PCV, 44 patients with nAMD and 51 age-, sex- matched healthy controls from RCSC as prescribed above.

The inclusion criteria were: 1) naïve PCV and nAMD not treated with anti-vascular endothelial growth factor (VEGF) at least within 1 year 2) No history of lipid-lowering medications. PCV was diagnosed by the fundus examination, fundus color photography, Swept Source optical coherent tomography (SS-OCT) and further confirmed by indocyanine green angiography (ICGA) according to EVEREST study report 2, modified EVEREST criteria (19–21) and the 2020 Asia-pacific eye image association PCV expert consensus of the working group (22). nAMD was diagnosed according to the “Expert consensus on macular degeneration” published in 2019 by Spaide et al. (23).

Exclusion criteria included those with 1) complicated with other fundus diseases such as macular hole, diabetic retinopathy, metastatic carcinoma, melanoma and others 2) history of serious systemic diseases including stroke and myocardial infarction within 6 months or not be able to tolerate eye examination 4) patients with history of metabolic disorders or on lipid lowering therapy. 5) subjects with one eye diagnosed with PCV and the other eye diagnosed with nAMD were also excluded.

Normal fundus control was defined as: 1) normal fundus in both eyes were evaluated by fundus ophthalmoscopy and OCT configuration 2) history of serious systemic diseases including stroke and myocardial infarction within 6 months or not be able to tolerate eye examination 3) patients with history of metabolic disorders or on lipid lowering therapy was excluded 4) subjects (with normal fundus in both eyes) with other systemic disorders were matched with the study groups when enrolled.

### Subgroup Grouping Criteria

The enrolled patients with PCV and nAMD were further categorized to the pachy-PCV/nAMD group or the non-pachy nAMD group respectively, according to the likelihood ratio test results determined in the same large cohort as we described previously (24). After controlling for age and diopter, the diagnostic cut off value range of pachy-choroid among 20-29 years old was 320-330 μm [likelihood ratio (LR): 1.17]; among 40-59 years old was 330-340μm (LR: 1.07); among 60 to 79 years old was 250-275 μm (LR: 1.07) and ≥ 80 years old was 200-225 μm (LR: 1.00).

### Eye Examination

The best-corrected visual acuity (BCVA), slit-lamp microscopic examination (SL-IE Slit Lamp Microscope, Topcon Co., Ltd, Tokyo, Japan), non-contact intraocular pressure (TX20 Automatic Non-contact Tonometer, Canon Co., Ltd, Tokyo, Japan), fundus photography (CR-1 non-mydriatic Fundus Camera, Canon Co., Ltd), OCT, fluorescent angiography and indocyanine green angiography (ICGA) were performed for all the participants. nAMD and PCV were diagnosed by OCT and indocyanine green angiography (ICGA) according to EVEREST study report 2, modified EVEREST criteria (20, 21) and the 2020 Asia-pacific eye image association PCV expert consensus of the working group (22).

### Lipoprotein Profiling and Routine Biochemical Examination

All participants underwent a standardized assessment of other risk factors including age, gender, duration of diabetes, duration of hypertension (HBP) and body mass index (BMI). The serum levels of TC, TG, LDL-C and HDL-C were tested by routine biochemical examination.

50 μL of serum was utilized to determine the level of apolipoproteins (A-I, A-II, B, C-II, C-III, E) determined by Luminex technology (Luminex 200™ liquid chip detector, Millipore, Boston, Massachusetts, USA), according to the manufacturer’s instructions.

Non-HDL-C, LDL/HDL, (TC-HDL)/HD, log (TG/HDL), apoA-I/apoA-II, apoB/non-HDL-C, apoB/apoA-I, apoC-III/apoC-II/and apo E/apoC-II ratios were also calculated.

### Determination of the Cutoff Value for apoA-I/apoA-II, apoB/non-HDL-C, apoB/apoA-I, apoC-III/apoC-II/and apoE/apoC-II Ratios by Receiver Operating Characteristic Curve

The cut off values for the ratios of apoA-I/apoA-II, apoB/non-HDL-C, apoB/apoA-I, ApoC-III/apoC-II, apo E/apoC-II, LDL/HDL, (TC- HDL)/HDL and log (TG/HDL) were determined by ROC curve. The indicators with high sensitivity and specificity (with maximum Youden index) were selected as the cut off values on ROC curve. The control group as stated above in 2.1. The cut off value of the apoB/non-HDL-C ratio was 0.43 [Area under curve (AUC): 0.62, sensitivity= 0.68, specificity=0.60], the apoB/apoA-I ratio was 1.77 (AUC: 0.544, sensitivity = 0.16, specificity= 0.94) in patients with PCV, the cut off value for the apoB/non-HDL-C ratio was 0.45 (AUC:0.71, sensitivity = 0.78, specificity = 0.63) in patients with nAMD. The ratio values (apoA-I/apoA-II, ApoC-III/apoC-II and apo E/apoC-II) with lower sensitivity (AUC< 0.50, sensitivity and specificity <0.5) were not considered in this study.

### Sample Size Determination

Sample size was calculated by the Power Analysis and Sample Size software (PASS 2022, NCSS LLC, Utah, USA) based on our pilot study result. According to the pilot study results, the mean value of LDL-C (which required maximum numbers among all the biochemical parameters) in the study group was 3.32 ± 0.79, in the control group was 3.08 ± 0.87, the minimum number per arm (sample size) was 36 subjects to detect the difference between the four groups with the designed power (1- beta= 90%) at 95% confidence level (alpha=0.05) as we previously described [32]. We increased the sample size to above 40 to in each group considering the variability (even in normal controls) of apos.

### Statistical Analysis

SPSS software (SPSS, Inc. 25.0, Chicago, IL, USA) was utilized for the statistical analysis. Baseline parameters including the age and gender of participants, duration of diabetes and HBP, BMI, TG, TC, HDL-C, LDL-C, the serum levels of apolipoproteins, LDL/HDL, (TC- HDL)/HDL, log (TG/HDL), the ApoB/ApoA-I ratio and the ApoB/Non-HDL ratio were described by means ± standard deviation (mean ± SD). One-way analysis of variance (ANOVA) or the Kruskal–Wallis test was applied according to the data distribution for the group comparisons. ANOVA or the univariate logistic regression was performed to assess the association between the serum levels of lipids, apolipoproteins and the ratios of lipoproteins and apolipoproteins between different groups. Multivariate logistic regression models were applied to evaluate the effects of serum lipids or apolipoproteins among PCV, nAMD and normal control groups. The Odds ratio for group comparison was analyzed using the normal control as the reference, OR > 1 means the variable is an independent risk factor for the study group, OR < 1 means the variable is an independent protective factor for the disease. When the disease sub-group was used as the reference, OR > 1 means that higher level of the independent variable was found in the study group, whereas an OR < 1 means the higher level of the independent variable was in the reference group (25). Statistical significance was defined as *p*<0.05.

## Results

### Baseline Demographic and Clinical Characteristics

A total of 148 subjects (84 males and 64 females, aged 51-94 years old) including 53 patients with PCV (aged 54-81 yrs, 67.49 ± 7.67 yrs), 44 patients with nAMD (aged 51-94 yrs, 71.30 ± 9.46 yrs) and age-, gender-, duration of hypertension and type 2 diabetes mellitus matched 51 subjects with normal fundus in both eyes (aged 51-78 yrs, 66.04 ± 7.91 yrs) were recruited from the outpatient clinic of Beijing Tongren Hospital from September 2016 to September 2021 (**Table 1**).

**Table 1:** Baseline demographic characteristics and biochemical parameters in subjects with PCV, nAMD and control.

	Control	PCV	nAMD	*F/χ^2^*	*P* value
	(*N* = 51)	(*N* = 53)	(*N* = 44)		
Age, years (mean ± SD)	66.04 ± 7.91	67.49 ± 7.67	71.30 ± 9.46	5.44^b^	0.066
Gender, female(N) (%)	26(50.98)	23(43.40)	15(34.09)	2.75^c^	0.253
Duration of Hypertension (mean ± SD)	4.36 ± 9.40	4.82 ± 9.17	8.08 ± 12.43	3.91^b^	0.141
Duration of DM (mean ± SD)	0.03 ± 0.16	2.13 ± 5.27	2.19 ± 6.31	5.88^b^	0.053
BMI (mean ± SD)	23.82 ± 3.38	24.45 ± 3.22	24.68 ± 2.43	0.95^a^	0.390
TG, mmol/L (mean ± SD)	1.42 ± 0.66	1.26 ± 0.52	1.22 ± 0.64	3.09^b^	0.213
TC, mmol/L (mean ± SD)	5.10 ± 0.99	5.16 ± 0.96	4.60 ± 1.27	3.59^a^	0.030*
LDL-C, mmol/L (mean ± SD)	3.09 ± 0.86	3.33 ± 0.87	2.81 ± 0.96	3.52^a^	0.032*
HDL-C, mmol/L (mean ± SD)	1.37 ± 0.38	1.32 ± 0.31	1.25 ± 0.33	1.35^a^	0.262
LDL/HDL (mean ± SD)	2.40 ± 0.85	2.66 ± 0.95	2.32 ± 0.84	2.70^b^	0.259
(TC- HDL)/HDL (mean ± SD)	2.94 ± 1.07	3.08 ± 1.08	2.78 ± 1.03	1.13^a^	0.325
log (TG/HDL) (mean ± SD)	-0.01 ± 0.29	-0.04 ± 0.21	-0.04 ± 0.25	0.21^a^	0.815
apo A-I, mg/ml (mean ± SD)	0.61 ± 0.15	0.64 ± 0.20	0.63 ± 0.15	0.96^b^	0.618
apo C-III, mg/ml (mean ± SD)	0.28 ± 0.11	0.28 ± 0.13	0.27 ± 0.14	0.31^b^	0.857
apo E, mg/ml (mean ± SD)	0.03 ± 0.01	0.03 ± 0.01	0.03 ± 0.01	0.10^b^	0.952
apo A-II, mg/ml (mean ± SD)	0.41 ± 0.08	0.43 ± 0.12	0.42 ± 0.09	0.99^a^	0.373
apo B, mg/ml (mean ± SD)	1.51 ± 0.39	1.72 ± 0.47	1.56 ± 0.56	2.40^a^	0.095
apo C-II, mg/ml (mean ± SD)	0.14 ± 0.06	0.14 ± 0.07	0.13 ± 0.07	0.40^b^	0.820
apo B/non-HDL-C (mean ± SD)	0.41 ± 0.09	0.45 ± 0.11	0.51 ± 0.15	13.05^b^	0.001*
apo B/apo AI(mean ± SD)	2.62 ± 1.00	2.82 ± 1.15	2.58 ± 0.83	0.51^b^	0.774
non-HDL-C, mmol/L (mean ± SD)	3.74 ± 0.96	3.84 ± 0.93	3.35 ± 1.10	3.07^a^	0.049*

*Statistically significant: p ≤ 0.05. Mean ± SD was showed in the Table . According to the type of data and the data distribution, ^a^ one-way ANOVA analysis, Post-hoc Bonferroni’s statistic. ^b^ Kruskal–Wallis. ^c^ Chi-square test were applied.

SD, standard deviation; DM, diabetes milletus; BMI, body mass index; TG, triglycerides; TC, total cholesterol; LDL-C, low-density lipoprotein cholesterol; HDL-C, high density lipoproteincholesterol;apo, apolipoprotein; PCV, polypoidal choroidal vasculopathy; nAMD, neovascular age-related macular degeneration; N, normal fundus control.

There were no statistically significant differences in age or gender between the PCV, nAMD and control groups (*p_age_* =0.066, *p _gender_* =0.253). No statistically significance difference was found in the duration of DM, HBP and BMI among the PCV, nAMD and healthy control groups (*p _duration of DM_* =0.053, *p _duration of HBP_* =0.141, *p _BMI_* =0.390). Furthermore, serum level of TC, LDL, apoB/non-HDL, non-HDL-C was significant differences among the three groups (*p _TC_* =0.030, *p _LDL-C_* =0.032, *p _apoB/non-HDL-C_=*0.001*, p _non-HDL-C_=*0.049) by one-way ANOVA analysis or Kruskal–Wallis test (**Table 1**).

### Comparison of the PCV Group and nAMD Group by Univariate Logistic Regression Analysis

Univariate logistic regression model showed that in comparison with healthy controls, apoB was an independent risk factors for PCV with statistical difference (OR 3.31, 95% CI 1.23-8.92, *p*=0.018) (**Table 2**).

**Table 2:** Comparisons of the variables between PCV, nAMD and control groups by univariate logistic regression analysis.

(a) PCV vs. N^‡^	Factor	OR	95% CI	*p* value
	Gender, ref: male	1.31	(0.60, 2.85)	0.493
	Age	1.03	(0.98, 1.08)	0.310
	BMI	1.07	(0.94, 1.23)	0.300
	Duration of hypertension	1.01	(0.96, 1.05)	0.760
	Duration of DM	1.80	(0.64, 5.01)	0.264
	TG	0.63	(0.32, 1.24)	0.180
	TC	1.03	(0.69, 1.53)	0.894
	LDL-C	1.32	(0.84, 2.08)	0.228
	HDL-C	0.59	(0.19, 1.84)	0.362
	LDL/HDL	1.38	(0.88, 2.16)	0.156
	(TC- HDL)/HDL	1.17	(0.81, 1.68)	0.408
	log (TG/HDL), per 0.1	0.96	(0.82, 1.12)	0.607
	apo A-I, per 0.1	1.11	(0.89, 1.39)	0.354
	apo C-III, per 0.1	1.06	(0.77, 1.47)	0.717
	apo E, per 0.01	1.07	(0.76, 1.51)	0.711
	apo A-II, per 0.1	1.31	(0.88, 1.95)	0.182
	apo B	3.31	(1.23, 8.92)	0.018
	apo C-II, per 0.1	1.08	(0.60, 1.97)	0.794
	apo B/non-HDL-C, per 0.1	1.17	(0.83, 1.63)	0.368
	apo B/apo AI	1.21	(0.83, 1.76)	0.315
	non-HDL-C	1.11	(0.73, 1.68)	0.632
(b) nAMD vs. N^‡^	Factor	OR	95% CI	*p* value
	Gender, ref: male	1.87	(0.81, 4.32)	0.142
	Age	1.10	(1.04, 1.16)	0.001
	BMI	1.08	(0.94, 1.25)	0.260
	Duration of hypertension	1.04	(1.00, 1.08)	0.088
	Duration of DM	1.78	(0.64, 4.96)	0.273
	TG	0.61	(0.32, 1.18)	0.141
	TC	0.67	(0.46, 0.98)	0.036
	LDL-C	0.70	(0.44, 1.12)	0.137
	HDL-C	0.39	(0.11, 1.30)	0.124
	LDL/HDL	0.89	(0.55, 1.44)	0.627
	(TC- HDL)/HDL	0.87	(0.59, 1.29)	0.483
	log (TG/HDL), per 0.1	0.96	(0.83, 1.12)	0.597
	apo A-I, per 0.1	1.13	(0.86, 1.49)	0.387
	apo C-III, per 0.1	1.01	(0.74, 1.37)	0.962
	apo E, per 0.01	1.10	(0.81, 1.50)	0.529
	apo A-II, per 0.1	1.09	(0.71, 1.68)	0.699
	apo B	1.76	(0.78, 3.94)	0.172
	apo C-II, per 0.1	0.97	(0.49, 1.89)	0.918
	apo B/non-HDL-C, per 0.1	2.08	(1.35, 3.21)	0.001
	apo B/apo A-I	0.96	(0.61, 1.50)	0.843
	non-HDL-C	0.68	(0.45, 1.03)	0.071
(c) nAMD vs. PCV^‡^	Factor	OR	95% CI	*p* value
	Gender, ref: male	1.43	(0.62, 3.29)	0.404
	Age	1.07	(1.01, 1.13)	0.015
	BMI	1.01	(0.87, 1.18)	0.883
	Duration of hypertension	1.03	(0.99, 1.08)	0.143
	Duration of DM	0.99	(0.92, 1.06)	0.763
	TG	0.88	(0.43, 1.79)	0.727
	TC	0.65	(0.44, 0.95)	0.027
	LDL-C	0.55	(0.34, 0.89)	0.015
	HDL-C	0.60	(0.17, 2.11)	0.422
	LDL/HDL	0.64	(0.40, 1.04)	0.074
	(TC- HDL)/HDL	0.74	(0.50, 1.10)	0.139
	log (TG/HDL), per 0.1	0.99	(0.83, 1.19)	0.929
	apo A-I, per 0.1	0.98	(0.78, 1.24)	0.888
	apo C-III, per 0.1	0.97	(0.73, 1.28)	0.810
	apo E, per 0.01	1.05	(0.78, 1.41)	0.740
	apo A-II, per 0.1	0.86	(0.60, 1.24)	0.417
	apo B	0.83	(0.40, 1.73)	0.617
	apo C-II, per 0.1	0.91	(0.50, 1.64)	0.751
	apo B/non-HDL-C, per 0.1	1.50	(1.08, 2.08)	0.016
	apo B/apo A-I	0.77	(0.50, 1.19)	0.238
	non-HDL-C-C	0.62	(0.40, 0.95)	0.027

^*^Statistically significant: p ≤ 0.05. ^‡^ represents for the reference group.

ref: reference; OR, Odd ratio; CI, confidence interval; BMI, body mass index; DM, diabetic retinopathy; TG, triglycerides; TC, total cholesterol; LDL-C, low-density lipoprotein cholesterol; HDL-C, high density lipoprotein-cholesterol; apo, apolipoprotein; PCV, polypoidal choroidal vasculopathy; nAMD, neovascular age-related macular degeneration; N, normal fundus control.

Compared with healthy controls, univariate logistic regression analysis showed that age (OR=1.10, 95%CI 1.04-1.16, *p*=0.001), apo-B/non-HDL-C (per 0.1) were independent risk factors for nAMD (OR=2.08, 95% CI 1.35-3.21, *p*=0.001). Interestingly, TC was found to have a protective effect for patients with nAMD with statistical significance (OR=0.67, 95% CI 0.46-0.98, *p*=0.036) (**Table 2**).

In comparison with nAMD, univariable logistic regression analysis showed that the serum levels of TC, LDL-C and non-HDL-C were significantly higher and associated with increased risk for PCV (OR_TC_= 0.65, 95% CI 0.44-0.95, *p*=0.027, OR _LDL-C_ =0.55, 95% CI 0.34-0.89, *p*=0.015, OR _non-HDL-C_ 0.62 95% CI 0.40-0.95, *p*=0.027, respectively). In addition, age was significantly older in patients with nAMD patients compared with that with PCV patients (OR=1.07, 95% CI 1.01-1.13, *p*=0.015) and apo-B/non-HDL-C (per 0.1) were independent risk factors for nAMD compared to PCV patients (OR=1.50, 95% CI 1.08-2.08, *p*=0.016) (**Table 2**).

### Comparisons of the Subgroups of PCV and nAMD by Univariable Logistic Regression Analysis

To further explore the correlations of the serum levels of apolipoproteins and lipid profiles with PCV and nAMD, patients with PCV and nAMD were sub-grouped to pachy (eyes with pachychoroid) and non-pachy groups according to the criteria as we described previously. Serum levels of TG were significantly lower in the pachy-PCV patients when compared with healthy controls (OR_TG_ 0.21, 95% CI 0.06-0.80, *p*=0.022). The univariate logistic regression analysis indicated that serum levels of apoA-II (per 0.1mg/dl) and Apo-B were significantly higher in patients with non-pachy PCV (OR _apoA-II_ 1.76, 95% CI 1.08-2.86, *p*=0.024; OR _Apo-B_ 5.74, 95% CI 1.72-19.12, *p*=0.004 respectively) (**Table 3**).

**Table 3:** Comparisons of the variables between the sub-groups (pachy and non-pachy) of PCV and control group by univariate logistic regression analysis.

(c) pachy-PCV vs. N^‡^	Factor	OR	95% CI	*p* value
	Gender, ref: male	0.56	(0.19, 1.66)	0.295
	Age	0.99	(0.92, 1.06)	0.702
	BMI	1.09	(0.92, 1.28)	0.326
	Duration of hypertension	0.99	(0.93, 1.06)	0.74
	Duration of DM	2.01	(0.58, 6.94)	0.27
	TG	0.21	(0.06, 0.80)	0.022
	TC	0.94	(0.53, 1.69)	0.84
	LDL-C	1.35	(0.71, 2.56)	0.358
	HDL-C	0.37	(0.06, 2.23)	0.28
	LDL/HDL	1.53	(0.83, 2.81)	0.175
	(TC- HDL)/HDL	1.20	(0.72, 2.00)	0.48
	log (TG/HDL), per 0.1	0.88	(0.72, 1.09)	0.254
	apo A-I, per 0.1	0.98	(0.95, 1.02)	0.323
	apo C-III, per 0.1	0.71	(0.42, 1.19)	0.197
	apo E, per 0.01	0.82	(0.49, 1.38)	0.453
	apo A-II, per 0.1	0.78	(0.43, 1.42)	0.412
	apo B	1.44	(0.39, 5.30)	0.579
	apo C-II, per 0.1	0.64	(0.25, 1.65)	0.354
	apo B/non-HDL-C, per 0.1	0.79	(0.47, 1.31)	0.354
	apo B/apo A-I	1.42	(0.87, 2.33)	0.161
	non-HDL-C	1.06	(0.59, 1.92)	0.835
(b) non-pachy PCV vs. N^‡^	Factor	OR	95% CI	*p* value
	Gender, ref: male	0.86	(0.36, 2.04)	0.723
	Age	1.05	(0.99, 1.11)	0.128
	BMI	1.04	(0.90, 1.21)	0.566
	Duration of hypertension	1.01	(0.97, 1.06)	0.58
	Duration of DM	1.97	(0.51, 7.61)	0.326
	TG	0.93	(0.46, 1.89)	0.837
	TC	1.07	(0.70, 1.63)	0.769
	LDL-C	1.30	(0.80, 2.13)	0.295
	HDL-C	0.73	(0.22, 2.45)	0.612
	LDL/HDL	1.31	(0.80, 2.16)	0.281
	(TC- HDL)/HDL	1.15	(0.77, 1.71)	0.506
	log (TG/HDL), per 0.1	1.01	(0.86, 1.19)	0.924
	apo A-I, per 0.1	1.32	(0.99, 1.74)	0.055
	apo C-III, per 0.1	1.31	(0.89, 1.93)	0.17
	apo E, per 0.01	1.22	(0.83, 1.80)	0.319
	apo A-II, per 0.1	1.76	(1.08, 2.86)	0.024
	apo B	5.74	(1.72, 19.12)	0.004
	apo C-II, per 0.1	1.40	(0.70, 2.79)	0.336
	apo B/non-HDL-C, per 0.1	1.50	(0.98, 2.30)	0.064
	apo B/apo A-I	1.10	(0.72, 1.67)	0.657
	non-HDL-C	1.12	(0.72, 1.76)	0.615
(c) non-pachy PCV vs. pachy-PCV^‡^	Factor	OR	95% CI	*p* value
	Gender, ref: male	1.52	(0.48, 4.81)	0.473
	Age	1.06	(0.99, 1.15)	0.117
	BMI	0.95	(0.79, 1.15)	0.600
	Duration of hypertension	1.03	(0.96, 1.10)	0.456
	Duration of DM	0.93	(0.84, 1.03)	0.144
	TG	6.52	(1.29, 32.97)	0.023
	TC	1.14	(0.62, 2.08)	0.675
	LDL-C	1.00	(0.52, 1.90)	0.987
	HDL-C	1.91	(0.30, 12.36)	0.498
	LDL/HDL	0.87	(0.48, 1.60)	0.659
	(TC- HDL)/HDL	0.96	(0.57, 1.63)	0.889
	log (TG/HDL), per 0.1	1.23	(0.93, 1.63)	0.155
	apo A-I, per 0.1	1.41	(1.01, 1.98)	0.044
	apo C-III, per 0.1	0.99	(0.76, 1.30)	0.964
	apo E, per 0.01	1.46	(0.84, 2.52)	0.176
	apo A-II, per 0.1	1.88	(1.02, 3.44)	0.043
	apo B	0.28	(0.07, 1.20)	0.086
	apo C-II, per 0.1	1.88	(0.76, 4.66)	0.171
	apo B/non-HDL-C, per 0.1	1.52	(0.95, 2.43)	0.082
	apo B/apo A-I	0.79	(0.48, 1.30)	0.361
	non-HDL-C	1.07	(0.57, 2.00)	0.836

^*^Statistically significant: p ≤ 0.05. ^‡^ represents for the reference group.

ref, reference; OR, Odd ratio; CI, confidence interval; BMI, body mass index; DM, diabetic retinopathy; TG, triglycerides; TC, total cholesterol; LDL-C, low-density lipoprotein cholesterol; HDL-C, high density lipoprotein-cholesterol; apo, apolipoprotein; Pachy-PCV, polypoidal choroidal vasculopathy with pachy-choroid; Non-pachy PCV, polypoidal choroidal vasculopathy with non-pachy choroid; N, normal fundus control.

The serum levels of TG, apoA-I (per 0.1mg/dl) and apoA-II (per 0.1mg/dl) were significantly higher in non-pachy PCV than those in pachy-PCV (OR _TG_ 6.52, 95% CI 1.29-32.97, *p*=0.023; OR _apoA-I_1.41, 95% CI 1.01-1.98, *p*=0.044; OR _apoA-II_ 1.88, 95% CI 1.02-3.44, *p*=0.043, respectively) (**Table 3**).

Compared to the healthy control group, the level of TG, TC, LDL-C and non-HDL-C were significantly lower in the pachy-nAMD group (OR _TG_ 0.10, 95% CI 0.02-0.61, *p*=0.013; OR _TC_ 0.38, 95% CI 0.18-0.81, *p*=0.012; OR _LDL-C_ 0.40, 95% CI 0.17-0.97, *p*=0.044; OR _non-HDL-C_ 0.35, 95% CI 0.15-0.82, *p*=0.015, respectively). Additionally, the apo-B/non-HDL-C ratio (per 0.1) was significantly higher in the pachy-nAMD group than that in the healthy control group (OR _apo-B/non-HDL-C_ 2.25, 95% CI 1.21-4.19, *p*=0.011) (**Table 4**). Age and apo-B/non-HDL-C ratio (per 0.1) were also significantly higher in the non-pachy nAMD group than that in the healthy control group (OR _age_ 1.09, 95% CI 1.03-1.16, *p*=0.004; OR _apo-B/non-HDL-C_ 2.06, 95% CI 1.29-3.27, *p*=0.002, respectively) (**Table 4**). In comparison with the pachy-nAMD group, TG was significantly higher in the non-pachy nAMD group (OR _TG_ 8.85, 95% CI 1.22-64.32, *p*=0.031) (**Table 4**).

**Table 4:** Comparisons of the variables between the sub-groups (pachy- and nonpachy-) of nAMD and control groups by univariate logistic regression analysis.

(c) pachy-nAMD vs. N^‡^	Factor	OR	95% CI	*p* value
	Gender, ref: male	0.18	(0.04, 0.87)	0.033
	Age	1.04	(0.96, 1.13)	0.327
	BMI	1.15	(0.94, 1.41)	0.169
	Duration of hypertension	1.01	(0.94, 1.08)	0.830
	Duration of DM	1.73	(0.61, 4.85)	0.300
	TG	0.10	(0.02, 0.61)	0.013
	TC	0.38	(0.18, 0.81)	0.012
	LDL-C	0.40	(0.17, 0.97)	0.044
	HDL-C	0.50	(0.08, 3.04)	0.451
	LDL/HDL	0.67	(0.31, 1.46)	0.314
	(TC- HDL)/HDL	0.66	(0.35, 1.25)	0.204
	log (TG/HDL), per 0.1	0.81	(0.64, 1.04)	0.099
	apo A-I, per 0.1	1.14	(0.73, 1.79)	0.562
	apo C-III, per 0.1	0.93	(0.54, 1.61)	0.805
	apo E, per 0.01	1.17	(0.72, 1.92)	0.524
	apo A-II, per 0.1	0.63	(0.33, 1.20)	0.16
	apo B	0.96	(0.19, 4.79)	0.964
	apo C-II, per 0.1	0.87	(0.29, 2.58)	0.794
	apo B/non-HDL-C, per 0.1	2.25	(1.21, 4.19)	0.011
	apo B/apo A-I	0.71	(0.31, 1.60)	0.405
	non-HDL-C, per 0.1	0.35	(0.15, 0.82)	0.015
(b) non-pachy nAMD vs. N^‡^	Factor	OR	95% CI	*p* value
	Gender, ref:male	0.69	(0.28, 1.71)	0.427
	Age	1.09	(1.03, 1.16)	0.004
	BMI	1.07	(0.91, 1.25)	0.426
	Duration of hypertension	1.04	(1.00, 1.08)	0.079
	Duration of DM	1.89	(0.36, 9.98)	0.455
	TG	0.87	(0.44, 1.72)	0.686
	TC	0.76	(0.51, 1.13)	0.176
	LDL-C	0.82	(0.50, 1.34)	0.425
	HDL-C	0.35	(0.09, 1.37)	0.129
	LDL/HDL	0.99	(0.58, 1.69)	0.968
	(TC- HDL)/HDL	0.97	(0.63, 1.49)	0.879
	log (TG/HDL), per 0.1	1.03	(0.87, 1.21)	0.763
	apo A-I, per 0.1	1.12	(0.84, 1.50)	0.445
	apo C-III, per 0.1	1.04	(0.73, 1.48)	0.851
	apo E, per 0.01	1.10	(0.77, 1.57)	0.615
	apo A-II, per 0.1	1.47	(0.85, 2.53)	0.168
	apo B	0.48	(0.20, 1.16)	0.102
	apo C-II, per 0.1	1.01	(0.46, 2.18)	0.991
	apo B/non-HDL-C, per 0.1	2.06	(1.29, 3.27)	0.002
	apo B/apo A-I	1.05	(0.65, 1.68)	0.849
	non-HDL-C	0.81	(0.52, 1.25)	0.332
(c) non-pachy nAMD vs. pachy-nAMD^‡^	Factor	OR	95% CI	*p* value
	Gender, ref: male	3.97	(0.75, 21.04)	0.105
	Age	1.05	(0.97, 1.14)	0.203
	BMI	0.85	(0.63, 1.14)	0.280
	Duration of hypertension	1.04	(0.96, 1.12)	0.342
	Duration of DM	1.01	(0.90, 1.13)	0.904
	TG	8.85	(1.22, 64.32)	0.031
	TC	1.38	(0.80, 2.39)	0.242
	LDL-C	1.54	(0.75, 3.17)	0.236
	HDL-C	0.67	(0.09, 4.90)	0.697
	LDL/HDL	1.49	(0.65, 3.42)	0.352
	(TC- HDL)/HDL	1.51	(0.74, 3.09)	0.256
	log (TG/HDL), per 0.1	1.38	(0.99, 1.91)	0.057
	apo A-I, per 0.1	0.99	(0.62, 1.58)	0.977
	apo C-III, per 0.1	1.06	(0.69, 1.65)	0.783
	apo E, per 0.01	0.96	(0.63, 1.46)	0.833
	apo A-II, per 0.1	1.91	(0.91, 4.00)	0.088
	apo B	0.59	(0.18, 1.95)	0.391
	apo C-II, per 0.1	1.13	(0.40, 3.18)	0.821
	apo B/non-HDL-C, per 0.1	0.84	(0.53, 1.31)	0.434
	apo B/apo A-I	1.68	(0.62, 4.54)	0.304
	non-HDL-C	1.62	(0.85, 3.08)	0.146

^*^Statistically significant: p ≤ 0.05. ^‡^ represents for the reference group.

ref, reference; OR, Odd ratio; CI, confidence interval; BMI, body mass index; DM, diabetic retinopathy; TG, triglycerides; TC, total cholesterol; LDL-C, low-density lipoprotein cholesterol; HDL-C, high density lipoprotein-cholesterol; apo, apolipoprotein; Pachy-nAMD, Neovascular age-related macular degeneration with pachy-choroid; Non-pachy nAMD, Neovascular age-related macular degeneration with non-pachy choroid.

### Comparisons in the Lipid Profiles and Apolipoproteins Between PCV and nAMD by Multivariable Logistic Regression Analysis

After adjusting for age, gender, BMI and duration of HBP, the results suggested that apo-B was an independent risk factors for PCV when compared to normal control (OR _Apo-B_ 4.06, 95% CI 1.38-11.96, *p*=0.011) (**Table 5**). The analysis also showed that the apo-B/non-HDL-C ratio (per 0.1) was an independent risk factor for nAMD, which is consistent with the univariate logistic analysis (OR 1.80, 95% CI 1.12-2.90, *p*=0.015) (**Table 5**).

**Table 5:** Comparisons in the lipid profiles and apolipoproteins between PCV, nAMD and control by multivariable logistic regression analysis.

(a) PCV vs. N^‡^	Factor	OR	95% CI	*p* value
	Gender, ref: male	0.90	(0.37, 2.19)	0.816
	Age	1.00	(0.94, 1.06)	0.991
	BMI	1.03	(0.90, 1.18)	0.68
	Duration of hypertension	1.02	(0.97, 1.06)	0.496
	apo B	4.06	(1.38, 11.96)	0.011
(b) nAMD vs. N^‡^	Factor	OR	95% CI	*p* value
	Gender, ref: male	0.88	(0.29, 2.65)	0.823
	Age	1.08	(1.01, 1.15)	0.029
	BMI	1.07	(0.89, 1.28)	0.480
	Duration of hypertension	1.00	(0.95, 1.05)	0.962
	apo B/non-HDL-C, per 0.1	1.80	(1.12, 2.90)	0.015
	TC	0.95	(0.58, 1.56)	0.840
(c) nAMD vs. PCV^‡^	Factor	OR	95% CI	*p* value
	Gender, ref: male	0.53	(0.19, 1.44)	0.213
	Age	1.07	(1.01, 1.14)	0.033
	BMI	1.03	(0.86, 1.23)	0.777
	Duration of hypertension	1.01	(0.97, 1.07)	0.562
	apo B/non-HDL-C, per 0.1	1.35	(0.93, 1.97)	0.112
	Factor	OR	95% CI	*p* value
	Gender, ref: male	0.58	(0.21, 1.59)	0.287
	Age	1.06	(1.00, 1.13)	0.061
	BMI	1.03	(0.87, 1.23)	0.704
	Duration of hypertension	1.01	(0.97, 1.06)	0.664
	non-HDL-C	0.60	(0.35, 1.03)	0.062

^*^Statistically significant: p ≤ 0.05. ^‡^ represents for the reference group.

ref, reference; OR, Odd ratio; CI, confidence interval; BMI, body mass index; DM, diabetic retinopathy; TG, triglycerides; TC, total cholesterol; LDL-C, low-density lipoprotein cholesterol; HDL-C, high density lipoprotein-cholesterol; apo, apolipoprotein; PCV, polypoidal choroidal vasculopathy; nAMD, neovascular age-related macular degeneration.

The results indicated that age is a risk factor specifically for nAMD compared with PCV (OR _age_ 1.07, 95% CI 1.01-1.14, *p*=0.033) (**Table 5**).

### Comparisons in the Lipid Profiles and Apolipoproteins Between Subgroups of PCV and nAMD by Multivariable Logistic Regression Analysis

To identify specific lipid metabolic dysfunction biomarkers, after adjusting for age, gender, BMI and duration of HBP, we compared nAMD and PCV, pachy and non-pachy PCV and nAMD by multivariate logistic regression analysis.

LDL-C was found to be a specific risk factor for patients with pachy-PCV, whereas lower level of TG was found in non-pachy PCV patients (OR _LDL-C_ 3.44, 95% CI 1.13-10.47, *p*=0.030; OR _TG_ 0.04, 95% CI 0.00-0.37, *p*=0.005, respectively) (**Table 6**). Furthermore, significantly increased levels of apoA-II (per 0.1mg/dl) and apoB were associated with increased risk of non-pachy PCV (OR OR apoA-II 1.94, 95% CI 1.03-3.68, *p*=0.042; OR aopB 6.20, 95% CI 1.37-28.02, *p*=0.018, respectively) (**Table 6**). In addition, higher level of apo-B/non-HDL-C (per 0.1) was associated with higher risk for non-pachy PCV in comparison with pachy-PCV (OR _apo-B/non-HDL-C_ 2.64, 95% CI 1.05-6.59, *p*=0.038) (**Table 6**).

**Table 6:** Comparisons in the lipid profiles and apolipoproteins between the subgroups of PCV (pachy- and nonpachy-) and control by multivariable logistic regression analysis.

(a) pachy-PCV vs. N^‡^	Factor	OR	95% CI	*p* value
	Gender, ref: male	0.49	(0.10, 2.47)	0.387
	Age	0.92	(0.82, 1.03)	0.13
	BMI	1.15	(0.91, 1.44)	0.238
	Duration of hypertension	1.00	(0.93, 1.09)	0.939
	TG	0.04	(0.00, 0.37)	0.005
	LDL-C	3.44	(1.13, 10.47)	0.030
	apo B/non-HDL-C, per 0.1	1.92	(0.78, 4.74)	0.159
(b) non-pachy PCV vs. N^‡^	Factor	OR	95% CI	*p* value
	Gender, ref: male	0.74	(0.24, 2.34)	0.609
	Age	1.01	(0.94, 1.09)	0.701
	BMI	0.94	(0.78, 1.14)	0.519
	Duration of hypertension	1.03	(0.98, 1.09)	0.222
	apo A-II, per 0.1	1.94	(1.03, 3.68)	0.042
	apo B	6.20	(1.37, 28.02)	0.018
(c) non-pachy PCV vs. pachy-PCV^‡^	Factor	OR	95% CI	*p* value
	Genfer, ref:male	1.59	(0.39, 6.41)	0.518
	Age	1.07	(0.97, 1.18)	0.155
	BMI	1.02	(0.82, 1.26)	0.882
	Duration of hypertension	1.01	(0.94, 1.10)	0.715
	apo B/non-HDL-C, per 0.1	2.64	(1.05, 6.59)	0.038
	apo C-III, per 0.1	0.92	(0.66, 1.28)	0.611

^*^Statistically significant: p ≤ 0.05. ^‡^ represents for the reference group.

ref, reference; OR, Odd ratio; CI, confidence interval; BMI, body mass index; DM, diabetic retinopathy; TG, triglycerides; TC, total cholesterol; LDL-C, low-density lipoprotein cholesterol; HDL-C, high density lipoprotein-cholesterol; apo, apolipoprotein; Pachy-PCV, polypoidal choroidal vasculopathy with pachy-choroid; Non-pachy PCV, polypoidal choroidal vasculopathy with non-pachy choroid.

Compared with healthy controls, age and the apo-B/non-HDL-C ratio (per 0.1) were strong risk factors for pachy-nAMD (OR _age_ 1.22, 95% CI 1.01-1.47, *p*=0.036; OR _apo-B/non-HDL-C_ 2.78, 95% CI 1.16-6.68, *p*=0.022, respectively) (**Table 7**). The apo-B/non-HDL-C ratio (per 0.1) was specific risk factor for non-pachy nAMD (OR _apo-B/non-HDL-C_ 1.76, 95% CI 1.08-2.87, *p*=0.023) (**Table 7**). The serum levels of apoproteins showed no differences between pachy-nAMD and non-pachy nAMD patients (**Table 7**).

**Table 7:** Comparisons in the lipid profiles and apolipoproteins between the subgroups of nAMD (pachy- and nonpachy-) and control by multivariable logistic regression analysis.

(a) pachy nAMD vs. N^‡^	Factor	OR	95% CI	*p* value
	Gender, ref: male	0.35	(0.04, 3.43)	0.366
	Age	1.22	(1.01, 1.47)	0.036
	BMI	1.36	(0.92, 2.02)	0.121
	Duration of hypertension	0.94	(0.84, 1.06)	0.315
	apo B/non-HDL-C, per 0.1	2.78	(1.16, 6.68)	0.022
(b) pachy-pachy nAMD vs. N^‡^	**Factor**	OR	95% CI	*p* value
	Gender, ref: male	0.93	(0.29, 2.95)	0.904
	Age	1.06	(0.98, 1.13)	0.137
	BMI	1.02	(0.83, 1.25)	0.861
	Duration of hypertension	1.02	(0.97, 1.07)	0.536
	apo B/non-HDL-C, per 0.1	1.76	(1.08, 2.87)	0.023
(c) non-pachy nAMD vs. pachy-nAMD^‡^	**Factor**	OR	95% CI	*p* value
	Gender, ref: male	2.19	(0.29, 16.59)	0.449
	Age	0.96	(0.86, 1.07)	0.425
	BMI	0.74	(0.50, 1.10)	0.133
	Duration of hypertension	1.09	(0.98, 1.21)	0.113
	apo B/non-HDL-C, per 0.1	0.71	(0.41, 1.24)	0.232
	**Factor**	**OR**	**95% CI**	***p* value**
	Gender, ref: male	2.21	(0.289, 16.9)	0.444
	Age	0.96	(0.86, 1.07)	0.42
	BMI	0.73	(0.49, 1.09)	0.126
	Duration of hypertension	1.09	(0.98, 1.20)	0.113
	LDL/HDL	0.85	(0.32, 2.28)	0.745
	apo B/non-HDL-C, per 0.1	0.72	(0.41, 1.24)	0.231
	**Factor**	**OR**	**95% CI**	***p* value**
	Gender, ref: male	2.20	(0.29, 16.75)	0.447
	Age	0.96	(0.86, 1.07)	0.416
	BMI	0.74	(0.49, 1.10)	0.132
	Duration of hypertension	1.09	(0.98, 1.21)	0.11
	(TC- HDL)/HDL	0.92	(0.40, 2.11)	0.843
	apo B/non-HDL-C, per 0.1	0.72	(0.41, 1.24)	0.232
	**Factor**	OR	95% CI	*p* value
	Gender, ref: male	1.89	(0.24, 14.97)	0.545
	Age	0.99	(0.88, 1.11)	0.816
	BMI	0.73	(0.49, 1.10)	0.129
	Duration of hypertension	1.10	(0.97, 1.24)	0.136
	log (TG/HDL), per 0.1	36.17	(0.10, 13544.76)	0.235
	apo B/non-HDL-C, per 0.1	0.58	(0.27, 1.24)	0.163

^*^Statistically significant: p ≤ 0.05. ^‡^ represents for the reference group.

ref, reference; OR, Odd ratio; CI, confidence interval; BMI, body mass index; DM, diabetic retinopathy; TG, triglycerides; TC, total cholesterol; LDL-C, low-density lipoprotein cholesterol; HDL-C, high density lipoprotein-cholesterol; apo, apolipoprotein; Pachy-nAMD, Neovascular age-related macular degeneration with pachy-choroid; Non-pachy nAMD, Neovascular agerelated macular degeneration with non-pachy choroid.

## Discussion

In this study, we found that the apoB/non-HDL-C ratio was an independent risk factor for nAMD, whereas apoB was an independent risk factor for PCV. In addition, age was a specific risk factor for nAMD compared with PCV. In the subgroup analysis for PCV and nAMD, it was found that apoB/non-HDL was an independent risk factor for both pachy- and non-pachy nAMD compared with control. apoB/non-HDL-C was also an independent risk factor for non-pachy PCV, indicating that non-pachy PCV shares pathological similarities with nAMD. LDL-C was an independent risk factor for pachy-PCV. These results above indicated differential regulated lipid profiles and apolipoproteins contributed to the phenotypes of PCV and nAMD.

Increasing evidence indicates that dysfunction of lipid metabolism plays a vital role in the pathogenesis of PCV and nAMD. The reverse cholesterol transport pathway regulated by *ABCA1* gene has been implicated in the development of choroidal neovascularization (26) and basal laminal deposits in mice on high-fat diets (27, 28). Genetic studies demonstrated that genetic variants associated with high-density lipoprotein (HDL) pathway, such as *LIPC*, *CETP* and *APOE*, are associated with the pathogenesis of nAMD or PCV (12, 29).

In this study, we determined the differential levels of apolipoproteins in plasma, lipid profiles and their ratios, and reported the associations of apolipoprotein-mediated lipoproteins formation with PCV and nAMD. ApoB, especially apoB-100 is involved in lipid metabolism and is the main component of lipoproteins. ApoB provides structural integrity to lipoproteins and shields the water-repellent lipids at its center. Compared with LDL-C, apoB level has been shown to be a better indicator for cardiovascular disease (30). In this study, we found that apoB and LDL-C were independent risk factors for PCV, indicating PCV is a lipid dysfunction associated disease.

As we reported previously (31), the ratios of apolipoproteins or lipoproteins reflect two or three parameters respectively, and serve as better indicators to mirror the metabolic and clinical interactions between lipid fractions. In the current study, we have showed that apoB/non-HDL-C was an independent risk factor for nAMD, pachy-nAMD, non-pachy nAMD, and non-pachy PCV, after adjusting for age, sex, duration of hypertension, BMI, and lipoproteins by multivariable logistic analysis. ApoB-100 binds one VLDL, LDL and lipoprotein (a) (Lpa) to form the lipoprotein particle. Each apoB can only bind one atherosclerotic lipoprotein particle (VLDL, LDL-C, Lpa), thus the serum level of apoB represents the level of atherosclerotic lipoprotein particles. However, the quality of cholesterol carried by apoB varied greatly due to the cholesterol LDL particles (rich or deplete LDL particles) (30). On the other hand, non-HDL-C is the arithmetic sum of cholesterol in VLDL and LDL particles, equivalent to all atherosclerotic cholesterol in lipoproteins (30). ApoB and non-HDL-C have mirror effects on each other, because apoB represents all the atherogenic particles and non-HDL-C represents all the atherogenic cholesterol. Higher level of apoB/non-HDL-C implies that there are more cholesterol-deplete atherosclerotic lipoprotein particles, lower ratio of apoB/non-HDL-C indicated there are more cholesterol-rich atherosclerotic lipoprotein particles. The INTERHEART study have shown that the risk of cardiovascular disease was lower when serum level of apoB was less than non-HDL-C (cholesterol-rich in ApoB particles) (25). Other studies showed that the cholesterol-deplete apoB is also associated with obesity, blood glucose disorders and cardiovascular diseases. We therefore hypothesized that atherosclerosis caused by cholesterol deficiency may play a role in the pathogenesis of pachy-nAMD, non-pachy nAMD, and non-pachy PCV, indicating that non-pachy PCV shares pathological similarities with nAMD, which is highly correlated with age-related atherosclerosis.

We found that apoB/non-HDL-C was a distinct biomarker for nAMD and non-pachy PCV, indicating that 1) non-pachy PCV may share pathological similarity with nAMD 2)nAMD and non-PCV closely correlated with age-related atherosclerosis. A biomarker was defined as a “characteristic that is objectively measured and evaluated as an indication of normal biological processes, pathogenic processes, or pharmacologic responses to a therapeutic intervention” by the Biomarker Definition Working Group, supported by the U.S. National Institute of Health (32). A biomarker can be diagnostic, monitoring, safety, and susceptibility/risk biomarkers based on its main clinical applications (33). Serum cytokines including IL-1α, IL-1β, IL-4, IL-5, IL-10, IL-13, and IL-17 were found in patients with AMD, supporting that inflammation contributes to the pathogenesis of AMD. Furthermore, IK-17 and TNF-α are indicators for good responder to anti-VEGF therapy in patients with AMD (34). Serum lipid profile (forty-one discriminating metabolites) identified by mass spectrometry, especially the distinct lipid metabolism activating factor was found to be a distinct indicator for PCV (11). Differently expressed mRNAs including ENSG00000249572, miRNA has-miR-20a-5p in peripheral blood mononuclear cells found were to be predictors for good and poor responders to anti-VEGF therapy in patients with nAMD (35). Furthermore, OCT and OCT angiography characteristics have been used as biomarkers for diagnosis and treatment on PCV and nAMD (36). The height of the pigment epithelium detachment (PED) and reflectivity of the content of the PED may predict polypoidal lesions closure (36). Re-examining the baseline scans for an inverted U-shaped elevation in anti-VEGF resistant cases was helpful for detecting polypoidal lesions closure (33). These cellular, molecular and imaging biomarkers have not only deepened our understanding the pathogenesis of PCV and nAMD, but also provide insight to find novel therapeutic targets.

As another risk factor for atherosclerosis, TG was found to play a significant protective role in pachy-PCV, which was supported by a meta-analysis (included 9 studies). The results showed that increased TG level of 1 mmol/L would significantly reduce the risk of AMD (relative risk, 0.91; 95% CI, 0.87 to 0.94; I^2^ = 2.6%; *p* = 0.42) (37). Several other studies also reported that lower TG level in AMD patients (in comparison with normal control), representing a reverse association between TG and cardiovascular diseases (16, 38).TGs are main from of lipid ester and the main constituent of body fat, containing glycerol and three fatty acids. The underlying mechanism of its protective effects on AMD or PCV merits further *in vivo* or *in vitro* studies.

A limitation of this study is that it was a nested case-control study with relatively small sample size. Therefore, further longitudinal follow-up studies of large cohorts are warranted to gain insight into the underlying pathological mechanisms and the causal relationship between apos and PCV/nAMD. Furthermore, as this study only included the subjects without lipid lowering therapy, the subjects with severe hyperglycemia may also be excluded, this group of patients may have distinct phenotype of nAMD or PCV, it is interesting to investigate the correlations between the plasma lipid biomarkers for these patients. Furthermore, although we have demonstrated the difference of the dysregulated apolipoproteins and lipid profile in PCV and nAMD, the present study should also merit consideration in interpreting the findings, which need to be further validated with well-designed, prospective, large cohort clinical studies. Nevertheless, this study provides a basis for future studies in PCV and nAMD subtypes on molecular mechanisms related to lipid metabolism.

In summary, to our knowledge, this is the first study to investigate the associations between the dysfunction of lipid metabolism and the phenotypes of PCV, nAMD, pachy- and non-pachy PCV and nAMD. Our results indicated that different regulatory mechanisms of lipid metabolism are associated with distinct phenotypes of PCV and nAMD. Differentially regulated apolipoproteins and lipid profiles could be used as novel biomarkers for PCV and nAMD.

## Data Availability Statement

The original contributions presented in the study are included in the article/supplementary material. Further inquiries can be directed to the corresponding authors.

## Ethics Statement

The studies involving human participants were reviewed and approved by Ethics Committee of Beijing Tongren Hospital, Capital Medical University. The patients/participants provided their written informed consent to participate in this study.

## Author Contributions

Conceptualization, XZ; methodology, XZ and BQ; formal analysis, XZ and BQ; investigation, XZ and BQ; Statistical analysis: XZ, BQ, ZG, YW. writing—review and editing, XZ and BQ; funding acquisition, XZ subjects enrolment: BQ; YN; XC. All authors contributed to the article and approved the submitted version.

## Funding

This work was supported by the National Natural Science Foundation of China [Grant 81570850 and 82070988] and the Ministry of Science and Technology Foundation of China [Grant 2016YFC1305604].

## Conflict of Interest

The authors declare that the research was conducted in the absence of any commercial or financial relationships that could be construed as a potential conflict of interest.

## Publisher’s Note

All claims expressed in this article are solely those of the authors and do not necessarily represent those of their affiliated organizations, or those of the publisher, the editors and the reviewers. Any product that may be evaluated in this article, or claim that may be made by its manufacturer, is not guaranteed or endorsed by the publisher.

## Abbreviations

ABCA1, ATP-binding cassette transporter A1; Apo, apolipoproteins; ApoA, apolipoprotein A; ApoB, apolipoprotein B; ApoC, apolipoprotein C; ApoE, apolipoprotein E; AUC, area under curve; BCVA, best-corrected visual acuity; BMI, body mass index; CETP, cholesterol ester transfer protein; HBP, hypertension; HDL-C, high-density lipoprotein; ICGA, indocyanine green angiography; IQR, interquartile range; LDL-C, low-density lipoprotein; LIPC, hepatic lipase; Lpa, lipoprotein(a); LR, likelihood ratio; nAMD, neovascular age-related macular degeneration; Non-pachy nAMD, nAMD with non-pachy choroid; Non-pachy PCV, PCV with non-pachy choroid; OCT, optical coherence tomography; OR, Odd ratio; Pachy-nAMD, nAMD with pachy-choroid; Pachy-PCV, PCV with pachy-choroid; PCV, Polypoidal choroidal vasculopathy; PED, pigment epithelium detachment; RCT: reverse cholesterol transport pathway; ROC, receiver operating characteristic; SFCT, Subfoveal choroidal thickness; TC, total cholesterol; TG, Triglycerides.
